# Spectrophotometric determination of Cu(II) in soil and vegetable samples collected from Abraha Atsbeha, Tigray, Ethiopia using heterocyclic thiosemicarbazone

**DOI:** 10.1186/s40064-016-2848-3

**Published:** 2016-07-26

**Authors:** Daniel Admasu, Desam Nagarjuna Reddy, Kebede Nigussie Mekonnen

**Affiliations:** 1 Department of Chemistry, College of Natural and Computational Sciences, Mekelle University, P.O. Box 231, Mekelle, Ethiopia; 2 Ezana Mining Development Analytical Laboratory, PLC, P.O. Box 788, Mekelle, Ethiopia

**Keywords:** Spectrophotometry, 2-Acetylpyridine thiosemicarbazone (2-APT), 3-Acetylpyridine thiosemicarbazone (3-APT), Soil, Vegetables

## Abstract

Two selective and sensitive reagents, 2-acetylpyridine thiosemicarbazone (2-APT) and 3-acetylpyridine thiosemicarbazone (3-APT) were used for the spectrophotometric determination of Cu(II). Both reagents gave yellowish Cu(II) complex at a pH range of 8.0–10.0. Beer’s law was obeyed for Cu(II)–2-APT and Cu(II)–3-APT in the concentration range of 0.16–1.3 and 0.44–1.05 µg/mL, respectively. The molar absorptivity and of Cu(II)–2-APT and Cu(II)–3-APT were 2.14 × 10^4^ at 370 nm, and 6.7 × 10^3^ L/mol cm at 350 nm, respectively, while the Sandell’s sensitivity were 0.009 and 0.029 µg/cm^2^ in that order. The correlation coefficient of the standard curves of Cu(II)–2-APT and Cu(II)–3-APT were 0.999 and 0.998, respectively. The detection limit of the Cu(II)–2-APT and Cu(II)–3-APT methods were 0.053 and 0.147 µg/mL, respectively. The results demonstrated that the procedure is precise (relative standard deviation <2 %, n = 10). The method was tested for Cu(II) determination in soil and vegetable samples. Comparisons of the results with those obtained using a flame atomic absorption spectrophotometer for Cu(II) determination also tested the validity of the method using paired sample *t* test at the 0.05 level showing a good agreement between them.

## Background

Copper can be considered either essential or hazardous to life and plays a substantial role in the environment (Fu and Yuan 2007; Horstkotte et al. 2012; Gouda and Amin 2014; Tarighat 2016). As a micronutrient, copper is responsible for the proper functioning of several metalloenzymes and its deficiency reduces the activity of not only copper containing enzymes but also some enzymes that do not contain copper (Kamble et al. 2011; Tarighat 2016). The deficiency of copper results in different health problems like anaemia, hair kinky, Wilson disease and jaundice (Nalawade et al. 2015). Excessive intake of copper can cause accumulation especially in liver cells and cause hemolytic crisis and neurological disturbances (Horstkotte et al. 2012). Apart from the biological activity of copper, major portion of world’s production of it is used in electrical equipments, roof sheeting, bronze paints and also finds its applications in agriculture as micronutrient fertilizers, fungicides, and insecticides (Kamble et al. 2011). Thus the determination of trace amounts of copper in various media is becoming increasingly important.

Several analytical techniques have been used for determination of copper, including atomic absorption spectrometry, atomic emission spectrometry, electroanalytical techniques, spectrophotometry, inductive coupled plasma-emission spectrometry, inductive coupled plasma-mass spectrometry, flow injection diode array spectrophotometry and X-ray fluorescence spectrometry (Pinto et al. 2004; Kruanetr et al. 2008; Gouda and Amin 2014; Nalawade et al. 2015; Tarighat 2016). However, spectrophotometry methods are often preferred, as they involve inexpensive instrument, less labor-intensive, and provide comparable sensitivity when appropriate chromogenic reagents are available (Dalman et al. 2002; Fu and Yuan 2007; Sabel et al. 2010; Kamble et al. 2011).

Copper(II) forms chelate complex with many chromogenic reagents containing ‘N’, ‘O’ and ‘S’ donor atoms (Nalawade et al. 2015). Some representative examples are *S*,*S*′-bis(2-aminophenyl)oxalate (Nohut et al. 1999), naphthazarin (Chaisuksant et al. 2000), 3-{2-[2-(2-hydroxyimino-1-methyl-propylideneamino)-ethylamino]-ethyl-imino}-butan-2-one oxime (H2mdo) (Dalman et al. 2002), *p*-anisidine with *N*,*N*-dimethylaniline (DMA) (Ohno et al. 2003), 1-phenyl-1,2-propanedione-2-oxime thiosemicarbazone (PPDOT) (Reddy et al. 2003), di-2-pyridyl ketone benzoylhydrazone (dPKBH) (Pinto et al. 2004), 3,3,5,5-tetramethybenzidine (TMB) (Di et al. 2005), thiomichlersketone (TMK) (Fu and Yuan 2007), 1-(2-thiazolylazo)-2-naphthol (Niazi and Yazdanipour 2008), benzyloxybenzaldehyde-4-phenyl-3-thiosemicarbazone (Prathima et al. 2010), formazan dye Zincon (Sabel et al. 2010), 1-(2,4-dinitro aminophenyl)-4,4,6-trimethyl-1,4-dihydropyrimidine-2-thiol (Kamble et al. 2011), sodium diethyldithiocarbamate (Na-DDTC) (Uddin et al. 2013) and 2-amino-4-(m-tolylazo) pyridine-3-ol (ATAP) (Gouda and Amin 2014).

Thiosemicarbazones are basically Schiff bases obtained by the condensation of a thiosemicarbazide, prepared from an aryl, aralkyl, or alkyl isothiocyanate and hydrazine, with an aldehyde or ketone under ambient conditions (Klayman et al. 1979; Padhye and Kauffman 1985; Beraldo et al. 2000, 2001; Mendes et al. 2001; Lobana et al. 2009; Soares et al. 2012; Cobeljic et al. 2014). Thiosemicarbazone derivatives are of considerable interest due to their versatility as ligands bearing suitable donor atoms for coordination to metals with strong coordinating ability toward different metal ions (Parrilha et al. 2011, 2012; Singh and Singh 2015). They show a wide range of chemical properties depending on the parent aldehyde or ketone; in particular, if these are heterocyclic aromatic systems, their nature seems to enhance their activity (Kovala-Demertzi et al. 2005; Prathima et al. 2010).

The aim of this work was to develop a highly sensitive, efficient and direct spectrophotometric method for Cu(II) determination using chromogenic reagents containing a Schiff base. The conditions for the direct spectrophotometric determination of Cu(II) with 2-APT and 3-APT were described. Various factors influencing the sensitivity of the proposed method such as the pH and ranges of applicability of Beer’s law on the determination of Cu(II) were also studied. The method was applied to soil and vegetable samples (tomato, cabbage and spinach) as well and compared to standard method (F-AAS).

## Results and discussion

### Characterization of 2-ATP and 3-ATP

The 2-ATP and 3-ATP were characterized by IR, ^1^HNMR and MS. The IR spectrum of 2-APT exhibits absorption bands corresponding to υ (N–H, asym), υ (N–H, sym), υ (C–H, pyridine), υ (C=N, Schiff’s base), υ (C–C, pyridine), δ (C–H, aromatic ring), υ (N–H, primary amide), υ (C=S) and δ (C–H, aromatic ring) at 3373 (m), 3261 (m, br), 3183 (s), 1608 (s), 1501 (s), 1466 (s), 1244 (w), 1086 (m) and 783 (m) cm^−1^, respectively. Similarly, the 3-APT shows bands corresponding to υ (N–H, asym), υ (N–H, sym), υ (C–H, pyridine), υ (C=N, Schiff’s base), υ (C–C, pyridine), υ (C–H, aromatic ring), υ (N–H, primary amide), υ (C=S) and δ (C–H, aromatic ring) at 3434 (m), 3387 (m, br), 3270 (s), 1611 (s), 1504 (s), 1406 (s), 1104 (w), 1088 (m) and 704 (m) cm^−1^, respectively. These results were comparable with the one reported by Beraldo et al. (2000, 2001), Chan et al. (2010), Cobeljic et al. (2014), Manikandan et al. (2014), Singh and Singh (2015).

The ^1^H NMR spectra of free ligand, 2-APT showed signal at δ 6.8–7.9 ppm, characteristics of the protons of aromatic moieties (pyridine ring protons) of the ligand were observed as multiplets. The peak appeared at 2.4 ppm has been assigned to methyl group (Kowol et al. 2008; Manikandan et al. 2014). Similarly, the ^1^H NMR spectra of free ligand, 3-APT showed a signal at δ 7.1–8.6 ppm (multiplets), characteristics of the protons of aromatic moieties (pyridine ring protons) of the ligand, while the methyl group peak appeared at 1.1 ppm. The mass spectrum of 2-APT and 3-ATP shows signal at 195 (M + 1) corresponding to its molecular ion peak. The molecular formula of the reagent is C_8_H_10_N_4_S (M. Wt 194).

**Fig. 1:**
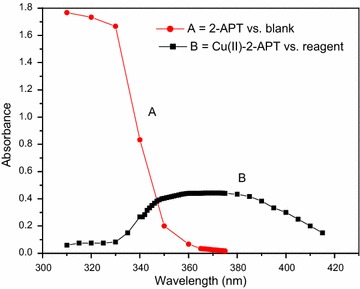
Absorption spectra of: **a** reagent (2-APT) versus blank; **b** Cu(II)–2-APT complex versus reagent, [Cu(II)] = 1 × 10^−4^ M, [2-APT] = 1 × 10^−2^ M, pH 9.0

**Fig. 2:**
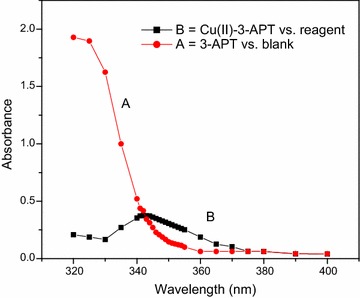
Absorption spectra of: **a** reagent (3-APT) versus blank; **b** Cu(II)–3-APT complex versus reagent, [Cu(II)] = 1 × 10^−3^ M, [3-APT] = 1 × 10^−2^ M, pH 9.0

**Fig. 3:**
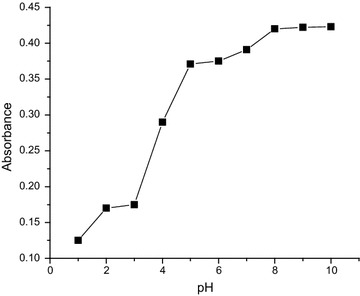
Effect of pH on the absorbance of Cu(II)–2-APT complex at a wavelength of 370 nm; [Cu(II)] = 1 × 10^−4^ M; [2-APT] = 1 × 10^−2^ M

**Fig. 4:**
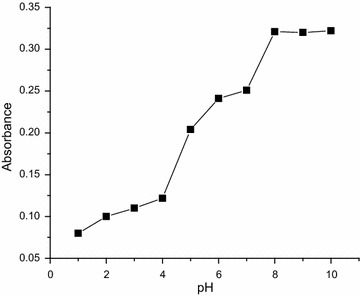
Effect of pH on the absorbance of Cu(II)–3-APT complex at a wavelength of 350 nm; [Cu (II)] = 1 × 10^−3^ M; [2-APT] = 1 × 10^−2^ M

The light yellow Cu(II)–2-APT and Cu(II)–3-APT complexes has a maximum absorbance at 370 and 350 nm respectively (Figs. 1, 2). These complexes are stable for 46 h. It is expected that the efficiency of the extraction process should mainly depend on the metal-extractant complex formation and the concentration of the extractable species (closely related to pH of the system). Therefore, the effect of the equilibrium pH on the extraction of Cu(II) was examined in the range from 1.0 to 10.0 for both methods. The results indicated that the complexes required alkaline (8.0–10.0) condition and hence pH of 9.0 was selected as the optimal condition for further experiments (Figs. 3, 4). The effects of reagent concentration on the absorbance of the complex were studied at λ_max_. The obtained results indicate that fivefold molar excess of reagent is required for full color development. Therefore further studies were carried out using at least fivefold molar excess of reagent to Cu(II).

### Beer’s law and sensitivity of Cu(II)–2-APT and Cu(II)–3-APT complexes

A calibration graph for the determination of copper was prepared under the optimum experimental conditions. The Cu(II)–2-APT system obeys Beer’s law in the concentration range of 0.16–1.3 µg/mL with the equation A_370_ = 0.66194C + 0.09596. The molar absorptivity and Sandell’s sensitivity of the method are 2.1 × 10^4^ L/mol cm and 0.009 µg/cm^2^ of Cu(II), respectively. The replicate (n = 10) analyses of a solution containing 1.0 µg/mL of Cu(II) gave 1.08 ± 0.0123 (%RSD = 1.14 %). The Cu(II)–3-APT system obeys Beer’s law in the concentration range of 0.44–1.05 µg/mL with the equation A_350_ = 0.51782C + 0.10188. The molar absorptivity and Sandell’s sensitivity of the method are 6.7 × 10^3^ L/mol cm and 0.029 µg/cm^2^ of Cu(II), respectively. The replicate (n = 10) analyses of a solution was 1.028 ± 0.01345 (%RSD = 1.31 %). The correlation coefficient values of the standard curves for Cu(II)–2-APT and Cu(II)–3-APT complexes were found to be 0.999 and 0.998, respectively, showing excellent linearity of the developed methods. The detection limit [expressed as 3 × standard deviation of blank (n = 10) divided by the slope of the calibration line] of the Cu(II)–2-APT and 3-APT methods are 0.053 and 0.147 µg/mL respectively. Comparing the two reagents, 2-APT is more sensitive than 3-APT for Cu(II) determination.

### Stoichiometry of Cu(II)–2-APT and Cu(II)–3-APT complexes

The spectrophotometric investigations of the Cu(II) complexes with 2-APT and 3-APT were conducted to obtain the composition of the complex. The composition of the complex was established by Job’s method of continuous variation (Huang et al. 2003; Mansour and Danielson 2012). The Job’s method of continuous variation has been used for determining the stoichiometry of coordination systems consisting of two interacting components (Huang et al. 2003). In this method the molar ratios are continuously varied while the total molar concentration of the two reactants is held constant. The binding stoichiometry is then determined from the ratio of the mole fractions of the two components found at the maximum of the curve (Huang et al. 2003). In the mole ratio method, varying amounts of ligand are added to a constant amount of metal ion. The absorbance of each solution is measured and plotted against the ratio of moles ligand/moles metal ions resulting in a curve having two straight line portions, while extrapolation gives the ratio of moles ligand/moles metal ion (Mansour and Danielson 2012). The application of Job’s method of continuous variation and mole ratio methods showed that 1:1 molar ratio is found in complex between Cu(II) and 2-APT or 3-APT reagent. Moreover, from Jobs method of continuous variation the stability constant of the complexes found to be 3.1 × 10^5^ and 8.4 × 10^5^, respectively.

### Analytical applications

In order to confirm the applicability of the proposed method, it has been applied to the determination of Cu(II) in vegetable and soil samples. The results for this study are presented in Tables 1 and 2. A flame atomic absorption spectrometry (F-AAS) method was used as a standard reference method and the results are also shown in Tables 1 and 2. The performance of the proposed method was compared with F-AAS method using student *t* test. Using paired sample *t* test at the 0.05 level, the developed methods are not significantly different with the standard method of F-AAS. Therefore, the results of the developed methods are in good agreement with the standard method. Moreover, comparing the results obtained from the two spectrophotometric reagents, the differences of the developed methods are not significantly different.

**Table 1:** Determination of Cu(II) (mean ± SD, n = 3) in vegetable samples

Vegetable	Amount of Cu(II) found (µg/g) by		
	F-AAS method	2-APT method	3-APT method
Tomato	5.4 ± 0.35	5.6 ± 0.41	6.0 ± 0.50
Cabbage	8.0 ± 0.26	8.7 ± 0.44	8.5 ± 0.46
Spinach	15 ± 0.4	16 ± 0.47	16.2 ± 0.39

**Table 2:** Determination of Cu(II) (mean ± SD, n = 3) in soil samples

Study area	Amount of Cu(II) found^a^ (µg/g) by		
	F-AAS method	2-APT method	3-APT method
Adi Desta	0.15 ± 0.01	0.19 ± 0.01	0.19 ± 0.01
Adi Nfas	0.88 ± 0.05	1.02 ± 0.07	1.02 ± 0.09
Adi Kulal	2.01 ± 0.08	2.40 ± 0.10	2.11 ± 0.01

The present method was also compared with other existing spectrophotometric methods in the literature (Chaisuksant et al. 2000; Dalman et al. 2002; Reddy et al. 2003; Di et al. 2005; Fu and Yuan 2007; Kamble et al. 2011; Nalawade et al. 2015) (Table 3). The developed methods are comparable with the reported methods with respect to the aforementioned analytical performances. Therefore, the proposed method could be used as an alternative method for copper determination in various media.

**Table 3:** Comparison of characteristic performance of the developed spectrophotometric methods for the determination of Cu(II) using thiosemicarbazones with some similar reported method

Name of the reagent	λ_max_ (nm)	Beer’s law range (ppm)	ε × 10^4^ (L/mol cm)	Applicability of the method	Reference
2-Acetylpyridine thiosemicarbazone (2-APT)	370	0.16–1.3	2.1	Soil and vegetables samples	This study
3-Acetylpyridine thiosemicarbazone (3-APT)	350	0.44–1.05	0.67	Soil and vegetables samples	This study
Naphthazarin (5,8-dihydroxy-1,4-naphthoquinone; Naph)	330	0.9–4.5	1.84	Alloy samples	Chaisuksant et al. (2000)
3-{2-[2-(2-Hydroxyimino-1-methyl-propylideneamino)-ethylamino]-ethyl-imino}-butan-2-one oxime (H2mdo)	570	0.2–225	0.16	Pharmaceutical and biological samples	Dalman et al. (2002)
1-Phenyl-1,2-propanedione-2-oxime thiosemicarbazone (PPDOT)	465	0.38–7.63	0.556	Edible oil and seed samples	Reddy et al. (2003)
3,3,5,5-Tetramethylbenzidine (TMB)	660	0.003–0.1	25.4	Multivitamin–multimineral tablet, river water and wastewater samples	Di et al. (2005)
Thiomichlersketone (TMK)	500	0–0.6	5.7	Water samples	Fu and Yuan (2007)
1-(2,4-Dinitro aminophenyl)-4,4,6-trimethyl-1,4-dihydropyrimidine-2-thiol [2,4-dinitro APTPT]	445, 645	10–80	0.087	Alloys, pharmaceuticals and biological samples	Kamble et al. (2011)
*N*′′,*N*′′′-bis[(E)-(4-Fluorophenyl) methylidene]thiocarbonohydrazide [bis(4-fluoroPM)TCH]	375	2.0–14	4.25	Alloys, food, pharmaceuticals and pesticide samples	Nalawade et al. (2015)

## Conclusion

In conclusion, 2-APT and 3-APT have been synthesized and characterized. The proposed ligands have been successfully applied as complexing agent to determine Cu(II) using spectrophotometry. The developed methods are practical and valuable for determination of copper. The results showed good agreement with the results obtained by F-AAS methods for soil and vegetable samples.

## Methods

### Sample collection

The soil samples were collected from Abraha Atsbeha village (13°50′N, 39°32′E), 58 km northeast of Mekelle, Tigray, Ethiopia. Three sampling spots in the village, namely Adi Desta, Adi Nfas and Adi Kula, were selected for soil sample collection while the vegetable samples were collected from the local market of the village. Topsoil composite samples were collected in the study area at a depth of 0–10 cm, with stones and foreign objects being removed by hand. They were kept in plastic bags, kept in ice-box, and once in the laboratory, oven-dried, sieved to < 2 mm, milled to the fine powder and stored in double-cup polyethylene bottles prior to analysis. The vegetables were rinsed in distilled water, air-dried, finely powdered and stored in double-cup polyethylene bottles prior to analysis.

### Chemicals

The chemicals used were 99 % *N*,*N*-dimethylformamide (DMF), 99.9 % dimethyl sulphoxide (DMSO), 98 % 2-acetylpyridine, 98 % 3-acetylpyridine, 99 % thiosemicarbazone, 37 % hydrochloric acid, 65 % nitric acid, 30 % hydrogen peroxide, 98 % sulphuric acid, ethanol, ammonium chloride, ammonium hydroxide, 99 % acetic acid, sodium acetate, 98 % copper sulfate pentahydrate (CuSO_4_·5H_2_O). All the chemicals were analytical grade reagents. All glassware were cleaned with 5 % HNO_3_.

### Instrumentation

The UV–Vis absorption spectra were recorded using Shimadzu 2450 double beam spectrophotometer at 800–200 nm range. An ELICO digital pH meter (Model LI-120) with combined glass electrode was used for measurement of pH. In order to characterize the synthesized reagent, the IR spectrum was recorded as KBr discs using Perkin-Elmer (spectrum 100) IR spectrophotometer in the 4000–200 cm^−1^ range. The ^1^HNMR spectra were taken at 400 MHz on a JEOL GSX-400 high resolution spectrometer at room temperature using tetramethylsilane as the internal standard. The mass spectra were recorded on Micro Mass VG-7070 H Mass spectrometer. The copper concentration was determined using Varian AA 240FS fast sequential atomic absorption spectrometer.

### Preparation of Cu(II) and buffer solutions

A stock solution of Cu(II) (1 mg/mL) was prepared by dissolving 3.93 g of CuSO_4_·5H_2_O in distilled water containing a few drops of conc. H_2_SO_4_. The solution was made up to 1 L and standardized by iodometry (Vogel 1989). This stock solution was further diluted, whenever necessary, with distilled water. The buffer solutions were prepared by mixing 1 M HCl and 1 M sodium acetate (pH 1–3), 0.2 M acetic acid and 0.2 M sodium acetate (pH 3.2–6.0), 1 M sodium acetate and 0.2 M acetic acid (pH 7.0), and 2 M ammonium hydroxide and 2 M ammonium chloride (pH 8.0–12.0). Suitable portions of these solutions were mixed to get the desired pH.

### Synthesis of Schiff base ligands and their respective metal complexes

**Scheme 1:**
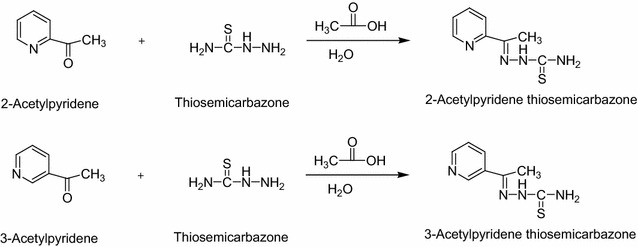
Preparation of 2-APT and 3-APT

The Schiff base, 2-APT was prepared using 3 mmol of 2-acetylpyridine in ethanol (10 mL) and mixed with equimolar amount of thiosemicarbazone in hot water (20 mL) (Scheme 1). The mixture was refluxed for 5 h and cooled to room temperature. The crystals obtained were subjected to filtration, washed with cold ethanol and dried (Manikandan et al. 2014). The same procedure was used for preparation of 3-APT using 3-acetylpyridine. A 1 × 10^−2^ M stock solution of each reagent was prepared by dissolving 0.049 g of the specific reagent with DMF and suitably diluted to get the required concentrations wherever necessary. For the synthesis of metal complexes, a hot ethanolic solution (25 mL) of free ligand (2 mmol) and a hot ethanolic solution (25 mL) of the metal salt (1 mmol) were refluxed for 4 h at 50 °C. After cooling to room temperature, the yellow solid was collected by filtration and washed sequentially with water and ethanol to give the desired product (West et al. 1993; Lobana et al. 2009; Chan et al. 2010; Prathima et al. 2010).

### Absorption spectra of reagent solutions and metal complexes

An aliquot of reagent (usually 1 mL of 1 × 10^−2^ M) solution was taken in a 25-mL volumetric flask containing 10 mL of buffer solution and made to the mark with distilled water. The absorbance of the reagent solution was measured against water blank. For measuring the absorption spectrum of complex, in a 25-mL volumetric flask, the metal complex in solution was prepared by taking 10 mL of buffer and suitable concentration of metal ion and reagent (tenfold molar excess to metal ion) solutions. The contents were diluted to the mark with distilled water and the absorbance was measured against the reagent blank.

### Effect of pH, concentration and time on the absorbance of the metal complexes

To examine the effect of pH, in a set of 25-mL volumetric flasks, 10 mL of buffer (pH 3.0–10.0) solution, constant amount of metal ion and reagent (usually tenfold molar excess to metal ion) solution were added and made to the mark with distilled water. The absorbance of each solution was measured at respective λ_max_ (370 and 350 nm, for 2-APT and 3-APT, respectively) against corresponding reagent blank. For finding optimum amount of reagent required known aliquot of reagent solution was taken in a set of 25-mL volumetric flasks, each containing 10 mL of buffer solutions and fixed amount of metal ion. The contents were diluted to the volume with distilled water and absorbance of the solution in each flask was measured at λ_max_ against corresponding reagent blank. Similarly, the absorbance of the colored complex solution was measured at λ_max_ against reagent blank at different time intervals so as to determine the stability of the complex and time interval required for full color development.

### Digestion procedure for soil and vegetable samples

The aqua regia method involving 6 mL of HNO_3_–HCl and 0.2 g samples using a conical beaker heated on a hot plate at 100 °C for 4 h was used to digest the soil samples (Hseu et al. 2002; Arain et al. 2008). About 0.5 g dried vegetable samples were weighed in Pyrex flasks and treated with HNO_3_–H_2_O_2_ (2:1) on hot plate at 100 °C for 3 h for decomposition of vegetable samples (Jalbani et al. 2007). The solutions were made up to 25 mL with distilled water and analyzed with F-AAS. The working calibration solutions were made up from 1000 mg/L certified standards and within the recommended linear ranges. The regression values (R^2^) of the calibration curve was >0.999. The concentration of Cu in the digested samples was determined at a wavelength of 324.8 nm using an air-acetylene flame. The analyses were conducted in triplicate and the results presented as mean ± SD.

### Determination of the stoichiometry of the complex

The composition of the Cu(II)–2-APT and Cu(II)–3-APT complexes were determined by Job’s continuous variation (Huang et al. 2003; Mansour and Danielson 2012) and mole ratio methods (Mansour and Danielson 2012). For Job’s method of continuous variation 10 mL of buffer solution, and equimolar solutions of Cu(II) and the reagent were prepared in a series of 25-mL volumetric flasks. The metal and reagent solutions were mixed in different proportions and diluted to the mark with distilled water. The absorbances were recorded at λ_max_, against the corresponding reagent blank. A plot between mole fraction of the metal ion and the absorbance was made. The stability constants of the complexes were also calculated. For mole ratio method 10 mL of buffer solution, constant amount of metal ion and known and varying aliquots of the reagent solutions were added in a series of 25-mL volumetric flasks. The contents of each flask were made to the mark with distilled water. The absorbances were recorded at λ_max_, against the corresponding reagent blank. The composition of the complex was ascertained from the plot between the absorbance and the volume of the reagent.

### Statistical analysis

All mathematical and statistical computations were made using Excel 2007 (Microsoft Office) and OriginPro 8.5.0 SR1 (OriginLab Corporation, USA). Data were reported as mean ± SD. Student *t* test was used for comparison of the developed method with standard method.
